# Risk factors for contralateral patent processus vaginalis determined by transinguinal laparoscopic examination

**DOI:** 10.3892/etm.2014.2098

**Published:** 2014-12-01

**Authors:** DONG-GI LEE, YOUNG-SUK LEE, KWAN HYUN PARK, MINKI BAEK

**Affiliations:** 1 Department of Urology, Kyung Hee University School of Medicine, Seoul, Republic of Korea; 2 Samsung Changwon Hospital, Sungkyunkwan University School of Medicine, Changwon, Republic of Korea; 3 Seoul Samsung Urology Clinic/Gynecology Health Care Center, Ulsan, Republic of Korea; 4 Department of Urology, Samsung Medical Center, Sungkyunkwan University School of Medicine, Seoul, Republic of Korea

**Keywords:** hydrocele, inguinal hernia, contralateral, laparoscopy

## Abstract

Concurrent contralateral inguinal exploration in children with unilateral hernia or hydrocele is a subject of debate. The aim of the present study was to investigate the incidence of contralateral patent processus vaginalis (CPPV) using transinguinal laparoscopy (inguinoscopy). In addition, the risk factors of CPPV were evaluated in order to facilitate the selection of appropriate candidates for contralateral examination. A total of 119 patients who presented with unilateral hydrocele, inguinal hernia or cryptorchidism between 2001 and 2008 underwent inguinoscopy during the ipsilateral surgery. All data were collected prospectively. The incidence of CPPV was investigated and the risk factors affecting the presence of CPPV were analyzed. Among these patients, 29 individuals (24.4%) had CPPV confirmed by inguinoscopy. No surgical complications were observed during the inguinoscopy. Cases with suspicious ultrasound findings were at a higher risk of CPPV than cases with normal findings (odds ratio, 13.800; P=0.004). A history of contralateral disease was also found to be a significant risk factor (odds ratio, 4.008; P=0.019). The present study identified that the significant risk factors for CPPV were suspicious findings on ultrasound examination and a history of contralateral disease. Therefore, it is concluded that performing inguinoscopy in children with these risk factors is beneficial.

## Introduction

Concurrent contralateral inguinal exploration in children with unilateral hernia or hydrocele is a subject of considerable debate. In 1952, Duckett reported that a contralateral hernia was present in as many as 30% of children presenting with unilateral hernias (1). In 1955, Rothenberg and Barnett recommended prophylactic contralateral exploration in all children (2). However, a meta-analysis revealed that the risk of contralateral hernia is only 5.76% (3). In addition, a previous study by the present authors found that the incidence was 9.5% (4). Therefore, it is widely considered that contralateral groin exploration is not justified in children with unilateral disease due to the low incidence of contralateral hernia and the potential for operative complications.

To avoid unnecessary contralateral inguinal exploration, several preoperative diagnostic tools, such as physical examination and herniography, have been used. However, these tests have low accuracy rates and high false positive rates (5). In 1992, laparoscopy was introduced as a tool for the diagnosis of contralateral patent processus vaginalis (CPPV) (6). If CPPV is observed laparoscopically, the PPV can be repaired through a groin incision or laparoscopy. Transinguinal laparoscopy (inguinoscopy) has been shown to be a safe, accurate and effective method of evaluating the contralateral side (7). Regardless of the convenience and accuracy of laparoscopy, performing laparoscopy for all patients with unilateral hernia or hydrocele is unreasonable.

The aim of the present study was to investigate the incidence of CPPV using inguinoscopy. In order to aid the selection of appropriate candidates for contralateral examination, the risk factors for CPPV were evaluated.

## Patients and methods

### Patients

Patients who presented with a unilateral hydrocele, inguinal hernia or cryptorchidism between November 2001 and March 2008 were enrolled. All data were collected prospectively. The study protocol was approved by the Samsung Medical Center Institutional Review Board (Seoul, Korea), and written informed consent was obtained from the patient’s relatives. Clinically bilateral disease and patients older than 6 years were excluded. In total, 119 patients were enrolled. All patients underwent concurrent transinguinal laparoscopy (inguinoscopy) during the initial surgery. The case records were reviewed for each patient and the variables evaluated included age at surgery; the side affected by hydrocele, inguinal hernia or cryptorchidism; gestational age; and birth weight. To define the risk factors for CPPV, the presence of the following was also assessed: i) previous history of contralateral hydrocele or hernia, which meant that the patient had presented with contralateral hydrocele or hernia in the past but did not have clinical contralateral disease at the time of the surgery; ii) suspicion of contralateral hydrocele on physical examination, which meant the patient did not have contralateral disease clinically but had scanty fluid or sac-like materials within the scrotum on palpation; and iii) findings suspicious for contralateral hydrocele (scanty fluid collection within the scrotum) on ultrasound.

One surgeon performed all the surgeries and physical examinations. As the herniated contents of the inguinal hernia included not only peritoneal fluid but also intestine or omentum, it was postulated that the hernia sac was wider than the hydrocele sac. The presence of an ipsilateral inguinal hernia was evaluated as a risk factor for CPPV.

### Inguinoscopy

**Figure 1:**
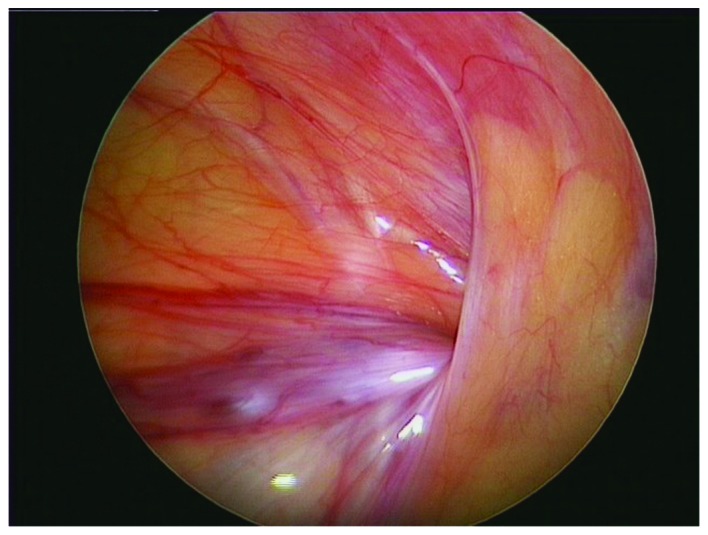
Patent processus vaginalis observed by transinguinal laparoscopic examination.

Inguinoscopy was performed during the ipsilateral surgery. The hernia sac was dissected free from the spermatic cord and traced to the internal inguinal ring. The sac was opened at the middle inguinal canal and a 4-mm reusable trocar was introduced through the hernia sac. CO_2_ gas was insufflated at a flow rate of 1 l/min to a pressure of 8–10 mmHg. A 45°-angled 3.3-mm endoscope was then introduced, and the contralateral internal inguinal ring was observed. Diagnostic criteria of CPPV (Fig. 1) on inguinoscopy were as follows: i) visible CPPV with passage of a guidewire >5 cm into the inguinal canal by >5 cm; ii) guidewire palpable in the contralateral scrotum; iii) bulging of the contralateral side during gas inflation; and iv) crepitus or air bubbles from the inguinal canal during scrotal manipulation.

### Statistical analysis

To evaluate each risk factor, univariate analysis was performed by Pearson Chi-square or Fisher’s exact tests. Logistic regression analysis was conducted to evaluate the association between risk factors and CPPV. Risk factors were reported with 95% confidence intervals. Data were analyzed using PASW^®^ Statistics, version 18.0 (SPSS, Inc., Chicago, IL, USA). A P-value <0.05 was considered to indicate a statistically significant result.

## Results

### Patient characteristics

The patients ranged in age from 8 to 72 months (median, 34 months). The median gestational age was 39.0 weeks (range, 26–41 weeks) and the mean birth weight was 3.2±0.50 kg (range, 2.2–4.3 kg). There were 62 children (52.1%) that presented with disease on the right, and 57 children (47.9%) that presented with disease on the left (Table I). Among these children, 87 (73.1%) presented with indirect inguinal hernia, 19 (16.0%) presented with cryptorchidism, and the remaining 13 (10.9%) presented with communicating hydrocele. Eight children (6.7%) had a history of preterm birth (gestational age <37 weeks), and five children (4.2%) had a history of low birth weight (birth weight <2.5 kg). There was history of a previous hydrocele or hernia on the contralateral side in 22 patients (18.5%), a suspicious physical examination in 46 patients (38.7%), and a suspicious ultrasound result in 8 patients (6.7%). Of the 119 patients, 29 patients (24.4%) had CPPV confirmed by inguinoscopy. No operative complications were observed during the inguinoscopy.

### Risk factors for CPPV

In the univariate analysis, cases with suspicious ultrasound findings had a higher incidence of CPPV than cases without suspicious findings (P=0.020). Age, preterm birth, lower birth weight, the affected side, a previous history of contralateral disease, a suspicious physical examination, an ipsilateral hernia, and ipsilateral cryptorchidism did not influence the incidence of CPPV (Table II). Multivariate analysis revealed that cases with suspicious ultrasound findings had a greater risk of CPPV than cases with normal findings (odds ratio, 13.800; P=0.004; Table III). A previous history of contralateral disease was also found to be a significant risk factor (odds ratio, 4.008; P=0.019).

## Discussion

There has been ongoing debate regarding concurrent contralateral inguinal exploration in children with unilateral inguinal hernia or hydrocele. The benefits of contralateral inguinal exploration include prevention of additional anesthesia and surgeries, minimizing parental and patient inconvenience, elimination of the possibility of incarceration, and reduced costs (8). By contrast, the dissenting opinion is that the true incidence of contralateral inguinal hernia is low (9). Recent studies have indicated that the incidence of metachronous contralateral inguinal hernia may be lower than originally expected. Meta-analyses have revealed an incidence rate of 5.76–7.2% (3,10,11). In addition, there is the potential for complications, including testicular atrophy and injury of the vas deferens or spermatic vessel, during the exploration (12).

As a result of this debate, there have been many efforts to identify appropriate candidates for exploration of the contralateral groin. The goal is to uncover the presence of CPPV. A PV is formed during the descent of the testis from the abdominal cavity to the scrotum (13). It is an extension of the peritoneum that is created by the pulling-down effect of the migrating testis on the lower abdominal peritoneal surface. Normally, the processus vaginalis is eradicated from the internal inguinal ring to the upper scrotum. However, if the processus vaginalis persists, peritoneal fluid is able to freely communicate with the scrotal limits of the processus, resulting in a communicating hydrocele. Inguinal hernias also develop due to the same anatomic defect that is seen in cases of a communicating hydrocele (13). The sac may contain small intestine, omentum, bladder or genital contents. As the majority of undescended testes, when examined, are found to have an accompanying patent processus, it is important that a cryptorchid testis is not missed when a child with a communicating hydrocele is examined (14).

The simplest and noninvasive method of diagnosis is physical examination using the silk glove sign. This involves detecting thickening and silkiness on palpating the spermatic cord as it crosses the pubic tubercle. Luo and Chao reported high sensitivity and specificity for this method (15). However, the results may vary depending on the examiner. Although the silk glove sign was not used in the present study, the physical examination by an experienced physician was not able to anticipate CPPV in the present study.

Laparoscopic examination is a recently introduced tool for the diagnosis of CPPV. Laparoscopy enables the direct visualization of anatomic defects of the contralateral internal inguinal ring. The sensitivity and specificity have been calculated to be 99.4 and 99.5%, respectively (5). Studies have shown that the additional time required for laparoscopic inspection is only 2–17 min (16). Laparoscopic evaluation may be performed using one of two methods. One method uses an umbilical approach (17); the other uses a transinguinal approach. The latter is performed more commonly and was the approach used in the present study. A trocar was inserted through the ipsilateral sac without a separate skin incision. Therefore, inguinoscopy may have a reduced risk of complications, as compared with an umbilical approach, particularly those associated with trocar placement. In the present study, no complications related to inguinoscopy were observed.

However, inguinoscopy has certain limitations. Since the peritoneal veil sometimes partially covers the internal inguinal ring, a direct view of the inguinal ring can be interrupted. The failure rates are reported as 3–8% (5). Although it was not always possible to observe the opened ring directly, bulging of the contralateral scrotum during gas inflation or air bubbles from the inguinal canal during scrotal manipulation are also evidence of PPV. Thus, it is considered that these diagnostic observations can overcome the limited visibility.

Controversy exists concerning whether the PPV should be closed. Not all PPVs develop into clinical inguinal hernia or hydrocele, and the PPV is sometimes obliterated spontaneously. Although ~80–94% of newborn infants have a PPV, ~60% will have disappeared by the time the child is 2 years old (18). In addition, the PPV may not cause the patient any problems. Autopsy data suggest that 15–30% of adults without a hernia have a PPV (19). The reported incidence of metachronous contralateral inguinal hernia is lower than the incidence of CPPV. The reported incidence of CPPV is 22–48% (7,20–27), which is similar to the incidence observed in the present study.

Laparoscopic examination is important for avoiding unnecessary inguinal exploration; however, the selection of appropriate patients is important in order to prevent unnecessary laparoscopy. In the present study, the significant risk factors for CPPV were found to be suspicious ultrasound findings and a history of contralateral disease. Therefore, it is concluded that it is beneficial to perform inguinoscopy in children with these risk factors. In addition to the risk factors identified in the present study, children with other well-known risk factors may also be candidates for inguinoscopy. As children with peritoneal dialysis, ventriculo-peritoneal shunts or ascites have increased intra-abdominal pressure, their risk of developing symptomatic disease is high. Children with contraindications for general anesthesia may benefit from inguinoscopy as it may avoid a second surgery.

In the present study, the incidence of CPPV was 24.4%. The significant risk factors for CPPV were suspicious ultrasound findings and a history of contralateral disease. Therefore, it is concluded that it is beneficial to perform inguinoscopy in children with these risk factors.

## Figures and Tables

**Table I:** Patient characteristics.

Variables	Value
Age at surgery (median, range, months)	34 (8–72)
Birth weight (mean ± SD, kg)	3.2±0.50
Gestational age (median, range, weeks)	39 (26–41)
Affected side [n (%)]	
Right	62 (52.1)
Left	57 (47.9)

**Table II:** Univariate analysis of risk factors for contralateral patent processus vaginalis.

Variables	N (%)	Contralateral patent processus vaginalis [n (%)]	P-value
Age			
≤2 years	34 (28.6)	6 (17.6)	0.280
>2 years	85 (71.4)	23 (27.1)	
Laterality			
Right	62 (52.1)	13 (21.0)	0.399
Left	57 (47.9)	16 (28.1)	
Gestestional age			
<37 weeks	8 (6.7)	2 (25)	0.625^a^
≥37 weeks	111 (93.3)	27 (24.3)	
Birth weight			
<2.5 kg	5 (4.2)	2 (40)	0.594^a^
≥2.5 kg	114 (95.8)	27 (23.7)	
Previous history			
Yes	22 (18.5)	9 (40.9)	0.057
No	97 (81.5)	20 (20.6)	
Physical examination			
Abnormal	46 (38.7)	14 (30.4)	0.221
Normal	73 (61.3)	15 (20.5)	
Ipsilateral hernia			
Yes	19 (16.0)	2 (10.5)	0.154^a^
No	100 (84.0)	27 (27.0)	
Ipsilateral undescended testis			
Yes	13 (10.9)	2 (15.4)	0.732^a^
No	106 (89.1)	27 (25.5)	
Ultrasound			
Abnormal	8 (6.7)	5 (62.5%)	0.020
Normal	111 (93.3)	24 (20.2)	

a Calculated by Fisher’s exact test.

**Table III:** Multivariate analysis of risk factors for contralateral patent processus vaginalis.

Risk factors	Odds ratio	95% confidence interval	P-value
Age ≤2 years	0.411	0.108–0.559	0.191
Right laterality	0.674	0.253–1.797	0.430
Preterm	1.327	0.139–12.671	0.806
Low birth weight	0.702	0.056–8.723	0.783
Previous history	4.008	1.254–12.805	0.019
Abnormal physical examination	2.839	0.992–8.123	0.052
Ipsilateral hernia	0.419	0.078–2.251	0.310
Ipsilateral undescended testis	0.583	0.092–3.694	0.567
Suspicious ultrasound findings	13.800	2.311–82.390	0.004
